# CTG Trinucleotide Repeat “Big Jumps”: Large Expansions, Small Mice

**DOI:** 10.1371/journal.pgen.0030052

**Published:** 2007-04-06

**Authors:** Mário Gomes-Pereira, Laurent Foiry, Annie Nicole, Aline Huguet, Claudine Junien, Arnold Munnich, Geneviève Gourdon

**Affiliations:** 1 INSERM, U781, Hôpital Necker Enfants Malades, Paris, France; 2 Université René Descartes Paris V, Paris, France; University of Minnesota, United States of America

## Abstract

Trinucleotide repeat expansions are the genetic cause of numerous human diseases, including fragile X mental retardation, Huntington disease, and myotonic dystrophy type 1. Disease severity and age of onset are critically linked to expansion size. Previous mouse models of repeat instability have not recreated large intergenerational expansions (“big jumps”), observed when the repeat is transmitted from one generation to the next, and have never attained the very large tract lengths possible in humans. Here, we describe dramatic intergenerational CTG•CAG repeat expansions of several hundred repeats in a transgenic mouse model of myotonic dystrophy type 1, resulting in increasingly severe phenotypic and molecular abnormalities. Homozygous mice carrying over 700 trinucleotide repeats on both alleles display severely reduced body size and splicing abnormalities, notably in the central nervous system. Our findings demonstrate that large intergenerational trinucleotide repeat expansions can be recreated in mice, and endorse the use of transgenic mouse models to refine our understanding of triplet repeat expansion and the resulting pathogenesis.

## Author Summary

Many neurological and/or neuromuscular diseases, such as myotonic dystrophy, Huntington disease, and fragile X mental retardation are caused by an increase in the size of a repeated DNA sequence within a specific gene. These repetitive DNA sequences are prone to expansion, increasing in size when transmitted from one generation to the next, which results in more severe symptoms and earlier age of onset. In myotonic dystrophy, the DNA repeat can undergo very large increments of several hundred units (frequently called “big jumps”), usually associated with the most severe clinical picture. Until now, big jumps have not been observed in mice carrying the disease mutation, leading to questions about the adequacy of mice to fully model DNA repeat instability. We now report that these large increments in the size of DNA repeats can occur in transgenic mice, resulting in animals that carry extremely large repeated sequences. These mice are remarkably small and display abnormalities in the metabolism of multiple messenger RNAs, notably in brain and muscle. Our findings strongly support the use of transgenic mice to resolve the complex dynamics of simple repetitive DNA sequences associated with human inherited diseases, and to investigate the molecular events that underlie the development of disease symptoms.

## Introduction

Trinucleotide DNA repeat expansion is the mutational cause of at least 14 neurological, neurodegenerative, and neuromuscular diseases in humans, including Huntington disease, many spinocerebellar ataxias, fragile X syndrome, and myotonic dystrophy type 1 (DM1). While expansion of other repeats can also be pathological, the majority of the triplet diseases are linked to instability of CTG•CAG repeat sequences [1,2]. The size of the inherited expansion is critically linked to the severity of the disease and the age of onset. The most surprising feature of this peculiar type of mutation is the marked tendency of the expanded repeat to further increase in size from one generation to the next, resulting in increased severity of the symptoms and earlier age of onset (anticipation) [3,4]. Anticipation is particularly evident in DM1 and is explained by the dramatic intergenerational instability of the CTG trinucleotide repeat: very large intergenerational expansions (“big jumps”) of several hundred repeats are frequently observed in DM1 families [5]. The repeat, normally ranging between 5–37 CTG, is expanded in DM1 patients, reaching up to 4,000 CTG in the most severe congenital form of the disease [6]. In addition, the disease-associated repeat expansion is also unstable in somatic tissues throughout the lifetime of the individual [7,8].

Modeling trinucleotide repeat instability in transgenic mice has been a challenge for more than a decade. Transgenic mice have been generated to provide in vivo models to study repeat instability in the germline and during somatic development, as well as to provide models of disease pathogenesis. Initial attempts using cDNA transgenes containing expanded CAG•CTG repeats failed to recreate repeat dynamics. A second generation of mice, carrying longer CAG•CTG tracts, or moderately sized expansions within their native genomic DNA context, reproduced intergenerational and somatic instability [9]. However, large intergenerational length increments of several hundred repeats have never been observed in these animals (Figure 1A). The smaller magnitude of intergenerational expansions in transgenic models raised the question of the adequacy of mice to fully model trinucleotide repeat instability.

**Figure 1:**
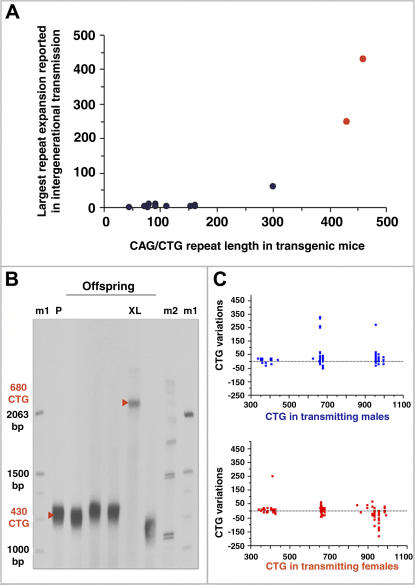
Big Jumps in Mice: Striking Intergenerational Trinucleotide Repeat Instability in Transgenic Mice (A) Transgenic mouse models of CAG•CTG instability previously generated exhibited modest intergenerational repeat expansions (blue dots). The largest expansions reported were smaller than 10 CAG•CTG repeats, with the exception of the DM300–328 line, in which intergenerational repeat gains reached +60 CTG [9]. Large CTG big jumps of several hundred repeats were recreated in a transgenic mouse model of DM1 carrying over 400 CTG repeats (red dots). To our knowledge, the repeat gains observed are the largest ever reported in transgenic models of unstable trinucleotide repeats. (B) Transmission of a very large expansion from a male parent (P) to its offspring. m1 and m2 indicate 100-bp and 250-bp molecular weight markers, respectively. (C) Intergenerational CTG size variation (yy axis) is represented for different CTG repeat lengths (xx axis) in male (top graph, blue dots) and female transmissions (bottom graph, red dots).

We report, to our knowledge for the first time, the occurrence in a transgenic mouse model of DM1 of CTG trinucleotide repeat big jumps in association with a strong phenotype and molecular abnormalities. This work demonstrates that large intergenerational mutations can accumulate in a mouse model, and provides a unique tool to further improve our understanding of the metabolism of unstable trinucleotide repeats associated with human disease.

## Results/Discussion

We have previously generated the DM300–328 transgenic mouse line, which carries over 300 CTG repeats embedded in 45 kb of human genomic sequence from the *DM1* locus. This line displays the highest frequency of intergenerational instability in mice reported until now (∼90% expansions in the offspring). Nevertheless, repeat changes never reached more than +60 CTG with a mean of +9 and +20 CTG for maternal and paternal transmissions, respectively [10]. Recently, two different mice derived from the DM300–328 line, carrying 430 and 460 CTG (male and female, respectively), transmitted big jumps of +250 CTG and +480 CTG in a single generation, producing offspring with 680 CTG and 940 CTG (Figure 1A and 1B). The male, carrying a remarkably long 680-CTG tract, transmitted larger expansions to 82% of its offspring (11% of transmissions resulted in repeat contraction). Among expansions, 11% consisted of further big jumps with a mean repeat gain of +290 CTG. We derived mouse sub-lines called “XL” for mice carrying ∼600–700 CTG and “XXL” for mice carrying ∼900-1000 CTG. Further large size gains were observed in these lines, and a male carrying 1,230 CTG was obtained, resulting from an additional +270-CTG expansion. Very large increments of CTG repeats seem to arise more frequently through male than female transmissions, with an overall frequency of 5% (Figure 1C).

We have previously shown that mice carrying 350–500 CTG display a mild phenotype, consistent with a *trans*-dominant effect of toxic RNA transcripts carrying CUG expansions [11–13]. A phenotype was only detected in homozygous mice for the low expressing transgene, suggesting a dose effect of the mutant RNA. Hemizygous mice for the very large expansions of 900 to 1,230 CTG did not show an obvious phenotype, indicating that the dramatic increase in CTG repeat size is not sufficient to overcome the low expression of the mutant RNA. However, homozygous mice carrying 700 CTG on one allele and 900 or 1,230 CTG on the other displayed a severe phenotype with very high mortality; 60% animals died before seven months of age. These animals exhibited severe growth retardation from birth (Figure 2) and marked splicing abnormalities in the central nervous system and muscle, through expression of toxic CUG-containing expanded myotonic dystrophy protein kinase *(DMPK)* transcripts (Figure 3). Notably, abnormal splicing of glutamate receptor, ionotropic, N-methyl D-aspartate 1 *(Grin1/Nmdar1)* and microtubule-associated protein tau *(Mapt*/*Tau)* transcripts was detected in the brain of homozygous transgenic mice carrying >700 CTG trinucleotide repeats within their human genomic context. In addition, a change in the distribution of muscleblind-like *(Mbnl1* and *Mbnl2)* isoforms either carrying or not carrying exon 7 was observed in the brain of these mice (Figure 3). To our knowledge, this is the first report of RNA splicing abnormalities in the central nervous system of a mouse model of DM1. The insulin receptor *(Insr)* and chloride channel 1 *(Clcn1)* transcripts also showed abnormal splicing patterns in skeletal muscle of the same animals (Figure 3).

**Figure 2:**
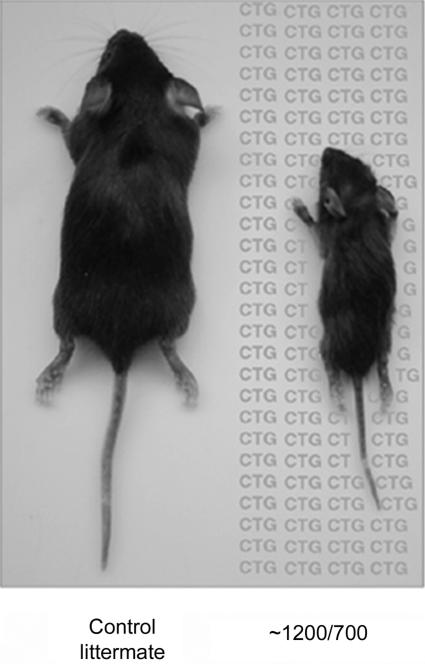
Body Size Reduction in Homozygous Mice Carrying Very Large CTG Repeat Expansions on Both Alleles Marked growth retardation in a 4-wk-old homozygous mouse carrying ∼1,230 and ∼700 CTG repeats compared to a control littermate.

**Figure 3:**
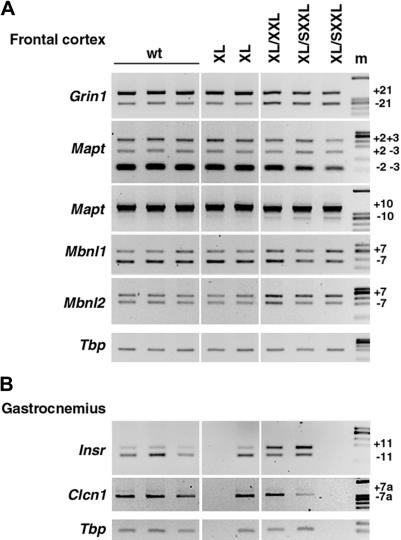
Abnormal RNA Splicing in Homozygous Mice Carrying over 600 CTG Trinucleotide Repeats on Both Alleles (A) Splicing abnormalities in the central nervous system. Abnormal splicing patterns were detected in the frontal cortex of 4-wk-old homozygous female mice (*n* = 3), when compared to wild-type (*n* = 3) and hemizygous female controls (*n* = 2) for mRNA transcripts of *Grin1* (favored exon 21 skipping), *Mapt* (altered splicing patterns of exons 2 and 3, and favored exon 10 skipping), *Mbnl1* (favored exon 7 inclusion), and *Mbnl2* (favored exon 7 inclusion). Splicing abnormalities were also observed in the hippocampus and striatum (unpublished data). (B) Splicing abnormalities in skeletal muscle. Reverse transcription-PCR experiments showed mRNA splicing defects for *Insr* (favored exon 11 inclusion) and *Clcn1* (modification in the ratio and quantity of +/– exon 7a isoforms) in the gastrocnemius muscle of 4-wk-old homozygous female mice (*n* = 2) when compared to wild-type (*n* = 3) and hemizygous female controls (*n* = 1). m, DNA molecular weight marker; wt, wild-type; XL, 600–700 CTG; XXL, 900-1000 CTG; SXXL, >1200 CTG.

In summary, we report remarkable, very large intergenerational changes in CTG repeat length in a mouse model of DM1. Striking expansions of several hundred repeats in a single generation resulted in the XL and XXL mouse lines, which carry the largest expansion ever observed in mice and display severe phenotypic and molecular abnormalities when homozygous for the very large repeat tract. The first two very large expansions arose in the same line but in mice raised in different animal facilities. The occurrence of these events in the line that we have the most extensively studied, is probably associated with a higher probability of getting very large expansions when the size of the repeat in the transmitting parent is larger, as Figure 1A appears to suggest. However, further mouse breeding strategies, especially with DNA repair–deficient lines, are needed to decipher the mechanisms involved in the formation of these very large expansions. The observation that hemizygous mice carrying very large repeats (over 1,000 CTG) do not display an obvious phenotype suggests that the expanded RNA dose is not sufficient to induce significant abnormalities. It is possible that the expression of the *DMPK* transgene might be lowered by the expanded CTG repeat, although this has not been described in DM1 patients. Furthermore, we cannot exclude a heterochromatinization spreading effect of the large repeat on the transgene and/or on the adjacent mouse genomic sequences. If only revealed by homozygous animals, this effect could contribute, at least partially, to the phenotype of mice carrying two very large CTG repeats. Future experiments will address these possibilities. Nevertheless, our results directly challenge the assumption that dramatic intergenerational triplet repeat expansions cannot be recreated in mouse models of unstable DNA. This important proof of principle provides an experimental foundation to further refine our understanding of the metabolism of disease-associated trinucleotide repeats. The lines generated in our study afford a unique tool to explore the complex dynamics of simple trinucleotide repeats, the increasing phenotypic severity through generations, as well as the molecular bases of RNA toxicity in disease pathogenesis.

## Materials and Methods

### Transgenic mice.

The transgenic mice used in this study carried 45 kb of human genomic DNA cloned from a DM1 patient as described by Seznec et al*.*[10]. Animals were bred onto a mixed background (C57BL6/129/OLA/FVB). Transgenic status was assayed by PCR using DMHR4 and DMHR5 oligonucleotide primers [10]. Tail DNA was amplified by PCR with oligonucleotide primers 101 and 102, and CTG repeat size was determined after electrophoresis of amplification products through a 4% polyacrylamide denaturing gel [10]. For the very large expansions (≥700 CTG), repeat size was measured after resolving the amplification products on a large 0.7% (w/v) agarose gel (20 × 35 cm). Animal care was performed according to institutional guidelines and approved by the Police Prefecture of Paris.

### RT-PCR analysis of alternative splicing.

Total RNA was extracted from microdissected brain regions using the RNeasy Mini Kit (Qiagen, http://www.qiagen.com). cDNA synthesis was primed with random hexamers. cDNA samples were treated with RNase A at 37 °C for 20 min. Most PCR amplifications were carried out for 21–26 cycles, within the linear range of amplification for each gene. The PCR amplifi-cation for *Mapt* exon 10 alternative splicing analysis was performed for 28 cycles, given the low abundance of the isoform lacking exon 10. The following oligonucleotide primers were used: *Grin1,* 5′-ATGCCCCTGCCACCCTCACTTTTG-3′ and 5′-GCAGCTGGCCCTCCTCCCTCTCA-3′; *Mapt* exons 2 and 3*,* 5′-ACTCTGCTCCAAGACCAAG-3′ and 5′-TGTCTCCGATGCCTGCTTC-3′; *Mapt* exon 10, 5′-CACCAAAATCCGGAGAACGA-3′ and 5′-CTATTTGCACCTTGCCACCT-3′; *Mbnl1* exon 7, 5′-GCTGCCCAATACCAGGTCAAC-3′ and 5′-TGGTGGGAGAAATGCTGTATGC-3′; *Mbnl2* exon 7, 5′-ACCGTAACCGTTTGTATGGATTAC-3′ and 5′-CTTTGGTAAGGGATGAAGAGCAC-3′; *Insr* exon 11, 5′-GAGGATTACCTGCACAACG-3′ and 5′-CACAATGGTAGAGGAGACG-3′; *Clcn1* exon 7a, 5′-CTTTGTAGCCAAGGTG-3′ and 5′-ACGGAACACAAAGGCACTGA-3′; and TATA box binding protein *(Tbp),* 5′-GGTGTGCACAGGAGCCAAGAGTG-3′ and 5′-AGCTACTGAACTGCTGGTGGGTC-3′. PCR products were resolved through 2.5% (w/v) agarose gels and stained with ethidium bromide.

## Supporting Information

### Accession Numbers

The National Center for Biotechnology Information GenBank (http://www.ncbi.nlm.nih.gov/Genbank) ID numbers for the genes discussed in this article are mouse *Clcn1* (118425), human *DMPK* (1760), mouse *Grin1* (14810), mouse *Insr* (16337), mouse *Mapt* (17762), mouse *Mbnl1* (56758), mouse *Mbnl2* (105559), and mouse *Tbp* (21379).

## Abbreviations

Clcn1: chloride channel 1
DM1: myotonic dystrophy type 1
Grin1: glutamate receptor ionotropic N-methyl D-aspartate 1
Insr: insulin receptor
Mapt: microtubule-associated protein tau
Mbnl: muscleblind-like
